# Effects of an Educational Intervention on Breast Self-Examination, Breast Cancer Prevention-Related Knowledge, and Healthy Lifestyles in Scholars from a Low-Income Area in Bogota, Colombia

**DOI:** 10.1007/s13187-016-1133-z

**Published:** 2016-11-04

**Authors:** A. M. Masso-Calderón, J. F. Meneses-Echávez, J. E. Correa-Bautista, A. Tovar-Cifuentes, P. A. Alba-Ramírez, C. E. Charry-Ángel

**Affiliations:** 1 Centro de Estudios en Medición de la Actividad Física (CEMA), Escuela de Medicina y Ciencias de la Salud, Universidad del Rosario, Bogotá, Colombia; 2 Norwegian Knowledge Centre for the Health Services in the Norwegian Institute of Public Health, PO Box 4404, Nydalen, N-0403 Oslo, Norway

**Keywords:** Breast cancer, Risk factor, Prevention

## Abstract

The aim of this study was to evaluate the effects of an educational intervention on breast self-examination, cancer prevention-related knowledge, practices, and behavior change in scholars from a low-income area in Bogota, Colombia. Uncontrolled trial was conducted in 155 scholars. Two educational sessions, 90 min each, were carried out in March 2015 according to the Colombian guidelines for educational communication in the framework of cancer control. All participants completed a self-reported questionnaire at pre- and post-intervention, as well as 1, 3, and 6 months after the intervention. Breast self-examination was practiced by 78.1% of the scholars, and the overall knowledge of breast cancer risk factors was poor. The educational intervention resulted in significant improvements on breast self-examination practice, the knowledge of the technique, and the knowledge of the main risk factors for breast cancer as well as the practice of physical activity and vegetable intake at 6 months follow-up. An educational intervention according to the Colombian guidelines for educational communication in the framework of cancer control improved the practice of breast self-examination, cancer prevention-related knowledge, as well as the practice of physical activity and vegetable consumption in scholars from a low-income area in Bogota, Colombia. Further randomized controlled studies are warranted.

## Introduction

Breast cancer is a serious public health problem, which has been recognized as the most common cancer in women worldwide [1]. In Colombia, breast cancer represents the third leading cause of death [2]. Breast self-examination (BSE) is related to self-awareness and self-care habits, associated with the facilitation of cancer screening [3]. There is no evidence about the effects of BSE on early detection of breast cancer [4]. Different organizations recommend regular self-examination in women over 20 years old in the promotion of self-awareness and risk control [5]. Nevertheless, most of women remain unaware about the technique of breast examination and the main risk factors for breast malignancies, especially of those related to lifestyle [6–8], although most of women can recognize hereditary factor as the main importance [9].

There is strong evidence that healthy lifestyles prevent breast cancer, as well as other type of cancers [10]. Physical activity exerts its effects on breast cancer prevention by modulating inflammatory markers [11], insulin-related factors [12], and other biological mechanisms [13]. Recently, Colditz and Bohlke [14] have reported that healthy lifestyles can reduce nearly half of breast cancer incidence annually; the control of physical inactivity could reduce 10% of such incidence. In addition, physically active young women can achieve risk reductions of around 25%, even more when active and healthy behaviors are adopted during youth and early adulthood [14]. As stated by McTiernan and colleagues, interventions that promote changes in lifestyle, including reduced alcohol consumption, reduced fat intake, and the practice of regular physical activity, can lower the risk of developing the disease and will have additional benefits by also lowering the risk of developing other noncommunicable diseases [15].

In light of this, educational interventions have been recommended as effective public actions in the integral control of breast cancer, especially among youth [16]. There is consistent evidence that educational interventions can improve both knowledge and practice of BSE in young women up to 6 and 12 months follow-up [17, 18]. Besides, educational interventions can also improve physical activity [19], as well as fruits and vegetable consumption in scholars [20, 21].

In the Colombian context, there are scarce evidences about educational interventions for breast cancer prevention, whereas the benefits of the national guidelines for educational communication in the framework of cancer control reaming is unknown [22]. The current agenda for cancer control in Colombia emphasizes on the need of community-based research for both detection and prevention [23]. The aim of this study was to evaluate the effects of an educational intervention on BSE, cancer prevention-related knowledge, and healthy lifestyles in a group of female scholars from a low-income area in Bogota, Colombia.

## Methods

### Design

The study design was uncontrolled trial with repeated measurements at 1, 3, and 6 months post-intervention.

### Participants

We selected a convenience sample of 155 female adolescents (10–20 years old), students of a public school in a low-income area in Bogota, Colombia. The purpose of study, interventions, and dates were explained in the classroom to the eligible students. Those who referred pregnancy, breastfeeding, or previous participation in a similar educational intervention were excluded.

### Ethics

Ethical approval to conduct this study was obtained from the ethics committee of the Our Lady of the Rosary University (Reference N. 306), after approval of the research protocol. Written informed consent was taken for each participant. Parents provided signed consent for each minor.

### Data Collection

#### Knowledge of Breast Cancer Risk Factors

We used the breast section of the Cancer Awareness Measure (CAM) [24, 25], which was developed and validated in 2007–2008 by the Cancer Research UK, the University College London, the King’s College, and Oxford University. The following question was used: Do you think that each of these can increase the chance of getting breast cancer? Responses were measured using the nominal scale of “yes” and “no.”

#### Breast Self-Examination

BSE practice and knowledge of the technique were measured using a validated questionnaire provided by the German multicentre DACH in the University of Düsseldorf, Germany [26]. For BSE, we asked the following: How often do you check your breasts? Responses were measured using the nominal scale of “yes” and “no.”

#### Healthy Lifestyle

We adapted the Spanish version of the Behavioral Risk Factor Surveillance System published by the Centers for Disease Control and Prevention (CDC) [27]. The following domains were evaluated: smoking, alcohol consumption, physical activity (>150 min/week), and fruits and vegetable intake. Weekly use/consumption was measured using the nominal scale of “yes” and “no.”

#### Questionnaire Validity

All modules were translated into Spanish by one researcher, and back translation was conducted and checked by one independent bilingual translator to ensure equivalence. The final version of the questionnaire was then piloted in a subgroup of 50 scholars who completed the tool and provided feedback regarding feasibility, clarity, and understanding. No lingual difficulties were identified.

The questionnaire was completed by all participants under supervision of the sports teacher, as suggested by the Cancer Research UK [25]. Cronbach’s alpha was used to assess the internal consistency, and Pearson’s correlation test was used to evaluate the test-retest reliability over a 7-day interval. Sensitivity to change is interpreted with the study results (i.e., changes scores).

### Educational Intervention

The educational intervention was developed in accordance with the national guidelines for educational communication in the framework of cancer control in Colombia [22], incorporating the use of clear language, flexible and understandable vocabulary. In order to facilitate the adherence and completion, the intervention contents were articulated to the school curriculum. The two 90 min educational sessions were carried out in March 2015 emphasizing on the normal breast, BSE, breast cancer risk factors and symptoms, the role of healthy lifestyles on breast cancer prevention, and methods for screening and early detection (Table 1). One researcher and teacher supervised both sessions and procedures. The pedagogical resources were videos, presentations, and open discussions. The students prepared various posters and placed them on the most crowded areas of the school. At the end of the workshop, each participant received a copy of the educational content provided. Follow-up assessments were undertaken at 1, 3, and 6 months after the intervention.

**Table 1:** Educational intervention for breast self-examination and breast cancer prevention

Educational intervention for breast self-examination and breast cancer prevention through healthy lifestyles: key components
Introduction to healthy lifestyles and cancer prevention
Review of the learning objectives
Normal breast (basic anatomy and physiology)
Breast self-examination
Breast cancer risk factors (i.e., modifiable and non-modifiable)
Symptoms of breast cancer
Early detection of breast cancer
Treatment options of breast cancer

### Statistical Analysis

The outcome of interest was the BSE practice, knowledge of breast cancer risk factors, and healthy lifestyles. The socio-demographic characteristics of scholars were described by using frequency, percentage, mean, and standard deviation. A multilevel regression model was used to estimate the change coefficients in the different scores with respect to pre-intervention measurements; each measurement was then assumed as a hierarchical source of variability. Alpha levels of *p* < 0.05 were considered as significant. The data were analyzed using the Stata version 22.0.

## Results

### Questionnaire Performance and Reliability

Cronbach’s alpha provided scores over 0.7 for all items in the questionnaire, which suggested a strong level of internal consistency. Similarly, Pearson’s correlation test showed strong test-retest reliability (over 0.72) for all items (*p* < 0.05) over a 7-day interval.

### Sociodemographic Characteristics of Study Participants

A total of 155 female scholars participated in the educational intervention and received the whole content. Their mean age was 13.72 years ± 1.9 standard deviations (SD) (range 10–20). All scholars are residents in a low-income area in south Bogota, Colombia. Only half of the participants live with both parents, and less than half of the parents have completed academic studies (Table 2).

**Table 2:** Sociodemographic characteristics of study participants

Characteristics	n	(%)
Age (years)		
<15	127	(81.9)
>15	28	(18.1)
Home composition		
Both parents	86	(55)
Only father	11	(7.1)
Only mother	32	(22.6)
Others	23	(14.8)
Father’s education		
None	30	(19.4)
High school	73	(47.1)
University	52	(33.5)
Mother’s education		
None	10	(6.5)
High school	78	(50.3)
University	67	(43.2)

### Effects of the Educational Intervention on BSE Knowledge, Practice, and Frequency

Baseline scores showed poor levels for these outcomes. Knowledge of the BSE technique showed a significant exponential increase across all measurements (*p* < 0.0001). Eighty percent of the scholars had never examined their breasts, and this rate showed a significant decrease of 67.8% (from 80.7 to 12.9%) at 6 months (*p* < 0.0001). Similar increases were also found for the frequency of BSE in all the categories evaluated. See Table 3.

**Table 3:** Effects of the educational intervention on BSE knowledge, practice, and frequency

Variable	Pre* *n* (%)	Post *n* (%) *p* value	1 month *n* (%) *p* value	3 months *n* (%) *p* value	6 months *n* (%) *p* value
BSE practice	41 (26.5%)	49 (5.1%) 0.273	62 (13%) 0.004**	61 (12%) 0.006**	120 (50%) <0.0001**
Knowledge of BSE technique	42 (27.1%)	94 (33%) <0.0001**	102 (38%) <0.0001**	121 (50%) <0.0001**	130 (56%) <0.0001**
BSE frequency**					
Never	125 (80.7%)	111 (71.7%)	100 (64.5%)	49 (31.6%)	20 (12.9%)
1–2 times/year	18 (11.6%)	14 (9%)	7 (4.50%)	0	0
3–10 times/year	9 (5.8%)	25 (16.1%)	38 (24.5%)	5 (3.2%)	14 (9%)
>10 times/year	3 (1.9%)	5 (3.2%)	10 (6.5%)	101 (65.2%)	121 (78.1%)

The percentages mean the score change with respect to pre-intervention values.

*Affirmative responses to each variable

**Statistically significant differences

### Effects of the Educational Intervention on Knowledge of Breast Cancer Risk Factors

Less than 10% of the participants recognized late-onset menopause, nulliparity, and the family history of cancer while only 37% of participants recognized sedentary behavior as a risk factor at pre-intervention. The scholars referred significant increases on knowledge of all risk factors up to 6 months after the intervention (Table 4).

**Table 4:** Effects of the educational intervention on knowledge of breast cancer risk factors

Breast cancer	Pre*	Post	1 month	3 month	6 month
risk factors	*n* (%)	*n* (%)	*n* (%)	*n* (%)	*n* (%)
		*p* value	*p* value	*p* value	*p* value
Age	89 (57.4%)	120 (77.4%)	127 (81.9%)	144 (92.9%)	145 (93.5%)
		(0.000)**	(0.000)**	(0.000)**	(0.000)**
Early menarche	34 (21.9%)	94 (60.6%)	106 (68.3%)	132 (85.1%)	142 (91.6%)
		(0.006)**	(0.000)**	(0.000)**	(0.000)**
Late pregnancy	33 (21.3%)	122 (78.7%)	123 (79.3%)	143 (92.2%)	148 (95.4%)
		(0.000)**	(0.000)**	(0.000)**	(0.000)**
Previous tumors	67 (43.2%)	99 (63.8%)	117 (75.4%)	147 (94.8%)	151 (97.4%)
		(0.000)**	(0.000)**	(0.000)**	(0.000)**
Family history of cancer	47 (9.7%)	77 (49.6%)	69 (44.5%)	110 (70.9%)	124 (80%)
		(0.104)	(0.718)	(0.000)**	(0.000)**
Nulliparity	5 (3.2%)	104 (67%)	105 (67.7%)	138 (89%)	145 (93.5%)
		(0.000)**	(0.000)**	(0.000)**	(0.000)**
Late-onset menopause	10 (6.5%)	101 (65.1%)	94 (60.6%)	129 (83%)	146 (94.1%)
		(0.000)**	(0.000)**	(0.000)**	(0.000)**
No breastfeeding	36 (23.2%)	105 (67.7)	108 (69.6%)	135 (87%)	145 (93.5%)
		(0.000)**	(0.000)**	(0.000)**	(0.000)**
Sedentarism	58 (37.4%)	123 (79.3%)	119 (76.7%)	118 (76.1%)	144 (92.9%)
		(0.000)**	(0.000)**	(0.000)**	(0.000)**
Low intake of fruits and vegetables	34 (21.9%)	93 (60%)	76 (49%)	121 (78%)	142 (91.6%)
		(0.000)**	(0.000)**	(0.000)**	(0.000)**
Sleep <7 h/day	15 (9.7%)	43 (27.7%)	53 (34.1%)	119 (76.7%)	144 (92.9%)
		(0.000)**	(0.000)**	(0.000)**	(0.000)**
Contraceptive pills	71 (45.8%)	116 (74.8%)	104 (67%)	133 (85.8%)	144 (92.9%)
		(0.000)**	(0.000)**	(0.000)**	(0.000)**

The percentages mean the score change with respect to pre-intervention values.

*Affirmative responses to each variable

**Statistically significant differences

### Effects of the Educational Intervention on Healthy Lifestyles for Breast Cancer Prevention

The educational intervention decreased weekly alcohol consumption at 6 months after the intervention (11% decrease). Significant improvements were observed on the practice of physical activity and vegetable consumption (*p* < 0.05). See Table 5.

**Table 5:** Effects of the educational intervention on healthy lifestyles for breast cancer prevention

Domains of lifestyle (Weekly use)	Pre* *n* (%)	Post *n* (%) *p* value	1 month *n* (%) *p* value	3 months *n* (%) *p* value	6 months *n* (%) *p* value
Smoking	6 (3.8%)	7 (4.5%)	9 (5.8%)	9 (5.8%)	7 (4.5%)
		(0.714)	(0.271)	(0.271)	(0.714)
Alcohol consumption	27 (17.4%)	22	(14.1%)	18 (11.6%)	18 (11.6%)
		(0.377)	(0.596)	(0.112)	(0.112)
Physical activity (>150 min/wk)	44 (28.3%)	49 (31.6%)	58 (37.4%)	105 (67.7%)	132 (85.1%)
		(0.061)	(0.000)	(0.000)	(0.000)
Fruit intake	119 (76.7%)	114 (73.5%)	114 (73.5%)	119 (76.7%)	118 (76.1%)
		(0.060)	(0.010)	(0.196)	(0.000)
Vegetable intake	70 (45.1%)	90 (58%)	68 (43.8%)	82 (52.9%)	127 (81.9%)
		(0.001)	(0.047)	(0.029)	(0.000)

The percentages mean the score change with respect to pre-intervention values.

*Affirmative responses to each variable

**Statistically significant differences

## Discussion

Our findings demonstrated an educational intervention improve the knowledge of BSE, its practice, the knowledge of the main risk factors for breast cancer, and healthy lifestyles in a group of female scholars up to 6 months follow-up.

### BSE Knowledge, Practice, and Frequency

We observed large improvements on BSE knowledge, practice, and frequency at 6 months follow-up. These findings are in line with those of recent studies carried out in Malaysia [17], Turkey [28], and Iran [29]. Nevertheless, it is important to consider some age differences in terms of applicability. This uncontrolled trial led to a complete adoption of monthly examination in the scholars.

### Knowledge of Breast Cancer Risk Factors

The knowledge of the Colombian female scholars was poor for most of the breast cancer risk factors and showed significant improvements after the educational intervention, which is similar to those reported in females from Malaysia [17], Iran [30], Egypt [31], and India [32]. These findings highlight the role of educational interventions in empowering young women in the battle against breast cancer.

### Healthy Lifestyles for Breast Cancer Prevention

In spite of a lack of statistical significance on some domains, we can state that this educational intervention improved healthy lifestyles in the scholars.

Alcohol consumption exhibited a small reduction at 6 months follow-up. Similar promising findings have been reported by the Project Toward No Drug Abuse [33] and the Project Northland [34] in high school students from the USA. Contradictory results have been found in Norway in junior high school students with a mean age of 13.5 years [35]. Further research has been desired by the latest Cochrane review [36].

Scholars referred an unexpected small increase (1%) in smoking at 6 months after the intervention. The Cochrane Tobacco Addiction Group has discussed similar results from 49 randomized controlled trials (over 140,000 school children) and concluded that prevention studies did not provide overall effect at 1 year or less, and multimodal interventions and those with an information-only approach were similarly ineffective [37]. However, a national level successful strategy “Ending the Tobacco Epidemic: A Tobacco Control Strategic Action Plan for the U.S. Department of Health and Human Services”, which combining all educational, clinical, regulatory, economic, and social initiatives, has shown decreases of up to 20% or more in some states [38].

We found exponential significant improvements in the practice of physical activity (>150 min/week) in the scholars. Similar findings have been communicated in a Cochrane review (44 studies involving 36,593 children and adolescents) which concluded that educational interventions combining printed educational materials and changes to the school curriculum that promote physical activity during school hours can lead to positive effects in increasing duration of physical activity from 5 to 45 min more per day and other outcomes such as the time spent watching television from 5 to 60 min less per day, and physical fitness [39]. Considering some context-related differences, we are able to recommend school-based interventions for the promotion of physically active behaviors aiming at noncommunicable disease prevention.

Our improvements in fruit and vegetable intake are similar to those published by Evans and colleagues [40] in 2012 summarizing evidence from 27 school-based programs involving 26,361 children, who reported increases of nearly 0.25 portions of fruit and vegetable daily intake. We did not evaluate the number of portions.

### Strengths and Weaknesses

This study is the first evidence in evaluating the Colombian guidelines for educational communication in the framework of cancer control. It is important to highlight the relevance of involving low-income groups in educational interventions since these individuals have limited access to most of the massive educational campaigns that take place in the country [23]. However, the lack of a random assignment and a control group preclude the statement of stronger conclusions about the effects of our educational intervention.

### Implications for Research

Further well-conducted randomized controlled trials can provide more rigorous evidence about the effects of educational interventions in improving female potential for breast cancer early detection and its prevention through healthy lifestyles. Further RCTs must incorporate strong sample sizes and adequate methods for allocation concealment, sequences generation, and outcome measurement. The use of objective measurements for lifestyle-related outcomes is also warranted.

### Implications for Practice

The results from this study are articulated with the priorities of the cancer risk control and prevention strategies of the Colombian Ministry of Health [23] and those from other international authorities. We recommend a detailed analysis of the applicability of our study findings since most of the current evidence comes from high-income countries.

## Conclusion

An educational intervention based on the Colombian guidelines for educational communication in the framework of cancer control improves BSE practice, breast cancer prevention-related knowledge, as well as the practice of physical activity and vegetable consumption in scholars from a low-income area in Bogota, Colombia. Further randomized controlled studies are warranted.
